# Clinical evidence of hyperbaric oxygen therapy for Alzheimer’s disease: a systematic review and meta-analysis of randomized controlled trials

**DOI:** 10.3389/fnagi.2024.1360148

**Published:** 2024-03-21

**Authors:** Guangyao Lin, Li Zhao, Jingyu Lin, Xuanling Li, Lianwei Xu

**Affiliations:** Department of Gynecology, Longhua Hospital, Shanghai University of Traditional Chinese Medicine, Shanghai, China

**Keywords:** hyperbaric oxygen, Alzheimer’s disease, cognition function, review, meta-analysis

## Abstract

**Objective:**

To evaluate the potential benefits of hyperbaric oxygen intervention on people with Alzheimer’s disease (AD) based on the existing randomized controlled trials (RCTs).

**Methods:**

A systematic search was conducted in nine databases until November 17, 2023, for RCTs assessing the effect of hyperbaric oxygen intervention for AD. The primary outcomes included Mini-Mental State Examination (MMSE), Alzheimer’s Disease Assessment Scale-Cognitive (ADAS-Cog), activities of daily living (ADL), and adverse events. All results were shown in forest plots, and sensitivity analysis was adopted to further verify the robustness of the pooled results.

**Results:**

A total of 11 RCTs recruiting 847 participants were included in this meta-analysis. Based on the pooled evidence, hyperbaric oxygen could remarkably ameliorate MMSE [MD = 3.08, 95%CI (2.56, 3.61), *p* < 0.00001], ADAS-Cog [MD = −4.53, 95%CI (−5.05, −4.00), *p* < 0.00001], ADL [MD = 10.12, 95%CI (4.46, 15.79), *p* = 0.0005], MDA levels [SMD = −2.83, 95%CI (−5.27, −0.38), *p* = 0.02], SOD levels [SMD = 2.12, 95%CI (1.10, 3.15), *p* < 0.0001], IL-1-β levels [SMD = −1.00, 95%CI (−1.48, −0.53), *p* < 0.0001], and TGF-β1 levels [MD = 4.87, 95%CI (3.98, 5.76), *p* < 0.00001] without adverse events [OR = 1.17, 95%CI (0.68, 2.03), *p* = 0.58] for people with AD. The pooled results were robust after checking by sensitivity analysis.

**Conclusion:**

These evidences suggest that hyperbaric oxygen is an effective and safe intervention for the treatment of AD. Further studies with more rigorous design will help to fully evaluate the clinical value of hyperbaric oxygen on cognition function in people with AD.

**Systematic review registration:**

https://www.crd.york.ac.uk, identifier CRD42023483726.

## 1 Introduction

In 195 countries worldwide, there has been a significant increase in the advanced-age population, mainly as a result of substantial population growth (Chang et al., 2019). By 2050, 20% of the global population will be represented by older adults (> 65 years), most of whom will be living in independently with a poor quality of life (Dogra et al., 2022). The overall rate of disability-adjusted life-years (DALYs) attributable to age-related burdens ranged from 137.8/1000 to 265.9/1000 in different countries between 1990 and 2017 (Chang et al., 2019). Notably, a growing number of health authorities across the globe are paying more attention to age-related burdens, such as Alzheimer’s disease (AD), which is one of the most common causes of DALYs (Beard et al., 2016; Deuschl et al., 2020). A recent statistic estimated that there are 6.2 million Americans over the age of 65 living with AD, and by 2060, this number will rise to 13.8 million (Alzheimer’s Dementia, 2021). Simultaneously, total spending on hospice services, wellness care, and long-term care for elderly with AD and people age 65 reached about $4,435.5 billion in 2021; besides, official death certificates reported 121,499 AD deaths in 2019, an increase of more than 145% between 2000 and 2019 (Alzheimer’s Dementia, 2021). Additionally, China, with the largest dementia population in the world, spent $248.71 billion on AD in 2020, and this cost will reach $1.89 trillion in 2050 (Jia et al., 2020). AD places a heavy burden on the global aging population and the public health systems; thereby, a great deal of effort is needed to accelerate the progression against this disease.

However, current treatment approaches have shown limited efficacy in ameliorating the disease progression (Joe and Ringman, 2019). Recently, a growing number of clinicians are striving to explore novel interventions to inform clinical practice in the treatment of AD. Hyperbaric oxygen therapy has been widely introduced in neurodegenerative disorders with supportive scientific evidence (Somaa, 2021; Mensah-Kane and Sumien, 2023). Although a previous meta-analysis investigating the clinical efficacy of hyperbaric oxygen for vascular dementia illustrated that hyperbaric oxygen treatment was strikingly associated with improvements in activities of daily living (ADL), Mini-Mental State Examination (MMSE), and an increase in the total efficacy rate, they failed to evaluate the clinical value of hyperbaric oxygen for patients with AD (You et al., 2019). Since AD accounts for 60% of dementia prevalence which comprises the first largest population of patients with dementia in China, but vascular dementia solely accounts for about 30% of dementia (Kalaria et al., 2008; Jia et al., 2020). Meanwhile, both AD and vascular dementia have different etiologies as well as diagnostic criteria, with the latter being diagnosed primarily after stroke. Subsequencely, the findings of previous meta-analysis might not be applicable to individual diagnosed with AD. Nevertheless, there is still a lack of evidence on the effectiveness of hyperbaric oxygen in improving behavioral and cognitive dysfunction in people with AD.

Therefore, the specific concern of this systematic review and meta-analysis was to quantitatively investigate the current existing randomized controlled trials (RCTs) of hyperbaric oxygen on ameliorating neuropsychiatric symptoms in people with AD. Moreover, we also examined the influence of hyperbaric oxygen on oxidative stress markers in blood, such as malondialdehyde (MDA) and superoxide dismutase (SOD), as well as inflammation cytokines, including interleukin-1(IL-1β) and transforming growth factor beta (TGF-β1).

## 2 Materials and methods

This study (PROSPERO registration No. CRD42023483726) was conducted following the preferred reporting program of the systematic review and meta-analysis (PRISMA) (Page et al., 2021).

### 2.1 Search strategy

All published records before November 17, 2023, were searched using six English databases (Cochrane Library, Web of Science, Scopus, PubMed, Sinomed, and EBSCO), and three Chinese databases (VIP Information, Wanfang, and China National Knowledge Infrastructure (CNKI)) for original articles. The search strategy comprises three components: clinical condition (Alzheimer’s disease, and dementia); intervention (hyperbaric oxygen), and study type (randomized clinical trial). Studies published in English or Chinese were independently checked via reviewing the title and abstract by two researchers (G.Y.L and L.Z). To ensure that all potential records could be identified as much as possible, a manual screen of reference lists from retrieved documents was conducted by two authors (G.Y.L and L.Z) as well. Eventual discrepancies were addressed through discussion with the corresponding author (LWX).

### 2.2 Inclusion and exclusion criteria

The inclusion criteria for this meta-analysis were as follows: (1) participant diagnosed with AD based on internationally recognized diagnostic criteria (Dubois et al., 2007; China Dementia and Cognitive Disorders Writing Group, Cognitive Impairment Disease Specialized Committee of Neurologists Branch of Chinese Physicians Association, 2018) (2) study adopted a parallel RCT design regardless of blinding; (3) clinical trial evaluated the effects of hyperbaric oxygen compared with a control group not receiving this intervention; (4) study published in English or Chinese.

Articles were excluded if they met the following criteria: (1) study failed to specify the diagnostic criteria of AD; (2) the type of study was not eligible for quantitative assessment (e.g., case report, study protocol, letter, or review); (3) patient belonged to subsequent dementia such as vascular dementia, Lewy body dementia, frontotemporal dementia, and Parkinson’s disease dementia; (4) study investigated the effectiveness of hyperbaric oxygen together with other medications like memantine and rivastigmine, whereas the control group did not adopt the same medications.

### 2.3 Data extraction and quality assessment

Data were independently extracted by two researchers (G.Y.L and J.Y.L) and collected in a predesigned form. The data extracted from each article included the author’s last name, publication year, number of groups and sample sizes in each group, mean age, duration of disease, treatment regimen, total duration of the intervention, and post-intervention results. The primary outcomes were MMSE, Alzheimer’s Disease Assessment Scale-Cognitive (ADAS-Cog), ADL, and adverse events. The secondary outcomes were MDA, SOD, IL-1β, and TGF-β1 in blood. Moreover, the quality of all included RCTs was evaluated with the help of the Cochrane Collaboration tool. Each record was graded as low, unclear risk, or high risk of bias regarding their methodological quality. Potential disagreements were resolve by consulting with the corresponding author (LWX), if necessary.

### 2.4 Statistical analysis

All analyses were performed utilizing Review Manager 5.3 software. The pooled effect of hyperbaric oxygen on AD was assessed by mean difference (MD) or standardized mean difference (SMD) with 95% confidence intervals (CIs) when analyzing continuous variables (e.g., MMSE, ADAS-Cog, and ADL); otherwise, odds ratio (OR) was adopted when estimating dichotomous variables (e.g., adverse events). The I^2^ statistics was employed to detect heterogeneity across studies. The random-effect model was performed when *I*^2^ > 50%, or a more appropriate fixed-effect model was applied. The level of significance was defined as *p* > 0.05. Furthermore, the robustness of the pooled results was verified through sensitivity analysis by excluding individual records.

## 3 Results

### 3.1 Included articles

**Figure 1:**
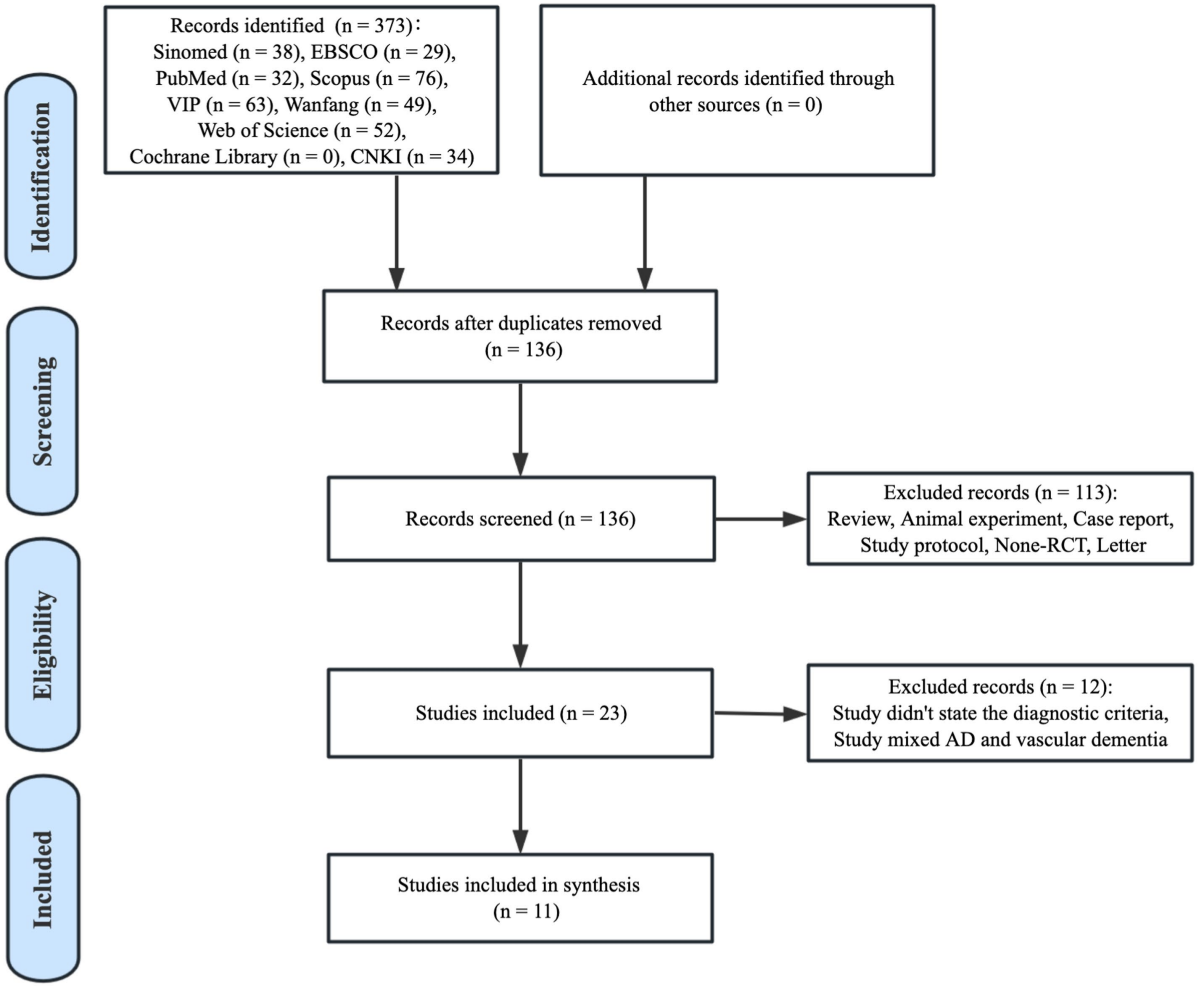
Paper selection flow chart.

The flow chart describing the literature search is depicted in the PRISMA figure (Figure 1). In total, 373 individual records were identified from nine databases. By screening the titles and abstracts, 237 duplicates were removed and 136 unique records remained. Whereas 113 studies, including reviews, animal experiments, case reports, study protocols, non-RCTs, and letters, were further excluded since they fulfilled our exclusion criteria. Subsequently, 23 relevant articles were considered in the full-text screening. Of these, 12 studies that failed to state the diagnostic criteria or mixed AD and vascular dementia were also excluded. Finally, 11 studies were assessed for qualitative synthesis.

### 3.2 Study characteristics

A total of 11 RCTs recruiting 847 participants were included in this meta-analysis. There were 433 and 414 people with AD in the trial group and control group, respectively. All included RCTs were published from 2007 to 2021 and carried out in China. The number of AD people in the 11 studies ranged from 43 to 98. Of the 11 studies, nine studies reported the age of patients, and five studies mentioned the duration of the disease. The treatment duration varied from 2 weeks to 6 months across studies. The general characteristics about the included studies are summarized in Table 1. Further, the details of hyperbaric oxygen therapy program were also presented in Supplementary Table S1.

**Table 1:** Study characteristics.

Study	Year	Total case (n)	Case (n)		Age (year)		Duration of disease		Treatment regimen		Treatment duration	Outcomes
			Trial group	Control group	Trial group	Control group	Trial group	Control group	Trial group	Control group		
Huang (2007)	2007	92	50	42	NA	NA	NA	NA	HBO + Rivastigmine	Rivastigmine	NA	①
Tian et al. (2007)	2007	92	50	42	71.8 ± 8.9	72.6 ± 9.1	NA	NA	HBO + Rivastigmine	Rivastigmine	NA	①
Yuan et al. (2010)	2010	43	23	20	70.8 ± 7.9	73.8 ± 8.5	NA	NA	HBO + Donepezil	Donepezil	12 weeks	①③
Liu et al. (2011)	2011	80	40	40	79.38 ± 6.539	78.08 ± 6.904	NA	NA	HBO + MXK capsules	MXK capsules	45 to 75 days	①③
Gao et al. (2017)	2017	60	30	30	72.5 ± 9.4	71.9 ± 8.7	2 to 11 years	2 to 13 years	HBO + Donepezil	Donepezil	2 months	②③④
Zhu et al. (2017)	2017	60	30	30	67.3 ± 4.1	68.5 ± 3.2	12.1 ± 5.3 months	11.3 ± 4.4 months	HBO	Wait-list	2 weeks	①②③④⑤⑥⑦⑧
Xu et al. (2019)	2019	72	36	36	68.92 ± 6.03	69.78 ± 4.38	NA	NA	HBO + Donepezil	Donepezil	2 months	①
Wang (2020)	2020	78	39	39	70.76 ± 5.43	70.42 ± 5.61	4.87 ± 0.62 years	5.34 ± 0.79 years	HBO + GDI	GDI	6 months	①④⑦
Zhang (2020)	2020	86	43	43	71.35 ± 5.73	71.52 ± 5.89	5.22 ± 1.19 years	5.42 ± 1.23 years	HBO + Memantine	Memantine	40 days	④⑤⑥
Wang et al. (2021)	2021	98	49	49	NA	NA	NA	NA	HBO + GBE	GBE	2 weeks	①②③④⑤⑥⑦⑧
Zhao et al. (2021)	2021	86	43	43	73.06 ± 2.55	73.28 ± 2.47	2.96 ± 0.27 years	3.01 ± 0.35 years	HBO + Donepezil	Donepezil	3 months	①③④

HBO, hyperbaric oxygen; NA, not applicable; GDI, *Ginkgo* leaf extract and dipyridamole injection; GBE, *Ginkgo biloba* leaf extract; MXK, Maixuekang; ①: MMSE ②: ADAS-Cog; ③: ADL; ④: adverse events; ⑤: MDA; ⑥: SOD; ⑦: IL-1β; ⑧: TGF-β1.

### 3.3 Risk of bias

**Figure 2:**
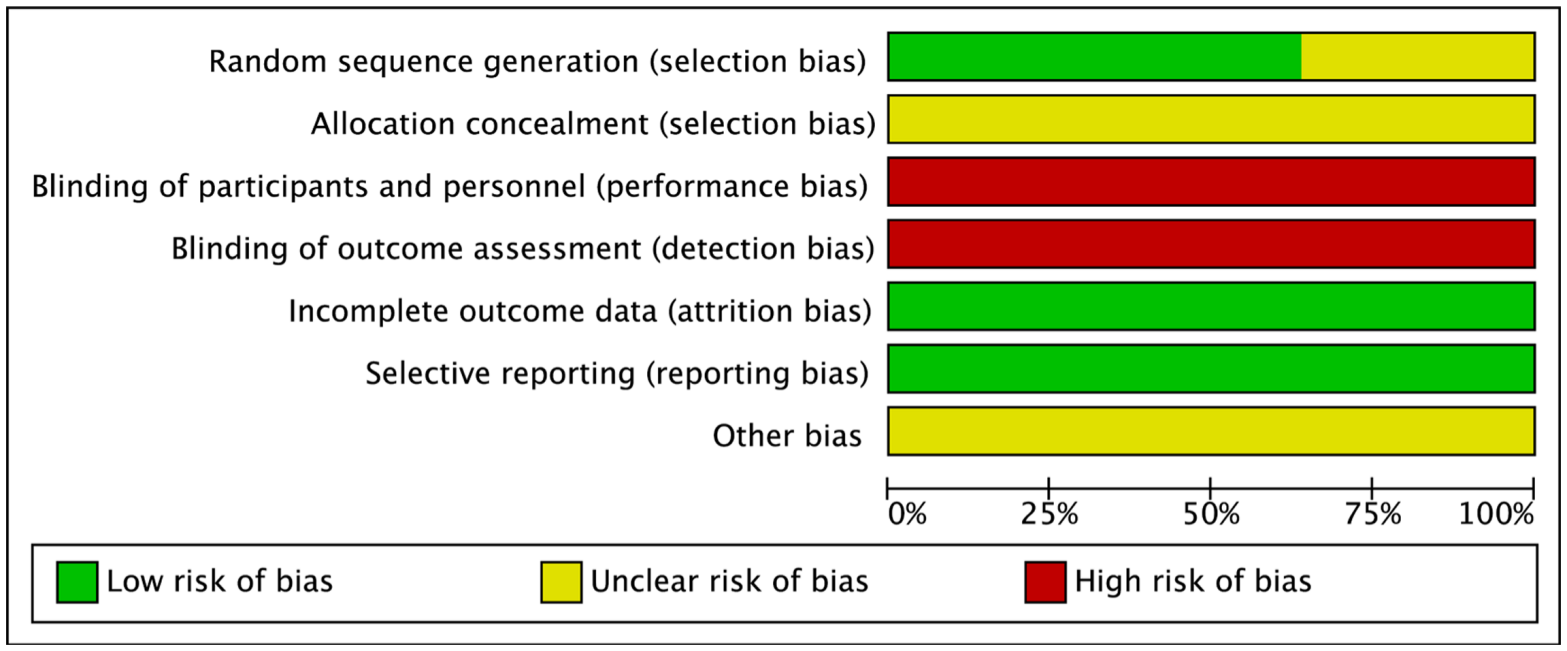
Risk of bias assessment.

The methodological quality of seven RCTs (Gao et al., 2017; Zhu et al., 2017; Xu et al., 2019; Wang, 2020; Zhang, 2020; Wang et al., 2021; Zhao et al., 2021) reported the procedure of division of AD patients in detail. Four studies (Huang, 2007; Tian et al., 2007; Yuan and Shi, 2010; Liu et al., 2011) solely described their randomization method as “random,” thereby were rated as having an unclear risk of bias in the domain of sequence generation. Also, all of these studies were judged as having an unclear risk of bias in the domain of allocation concealment as none of them provided information on this. Further, 11 RCTs did not achieve blinding throughout their study and thus were regarded to have a high risk of bias. Moreover, all included studies did not mention the details of incomplete outcomes as well as selective reporting and hence were rated as low risk of bias. However, underlying bias was not detected in all these studies. Collectively, the leading risk of bias lies in the blinding for this meta-analysis (Figure 2).

### 3.4 Outcome measurements

#### 3.4.1 Primary outcomes

**Figure 3:**
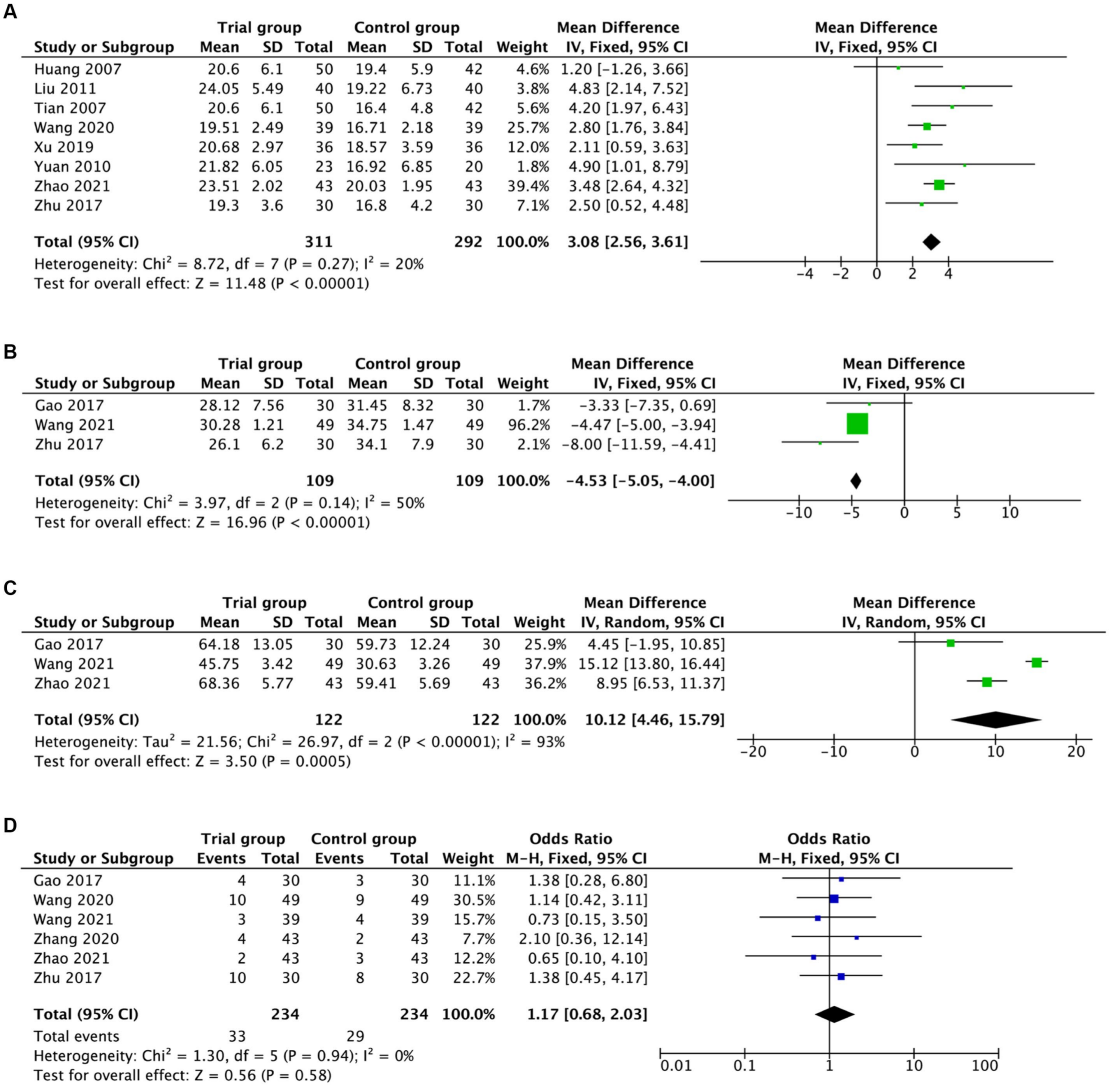
Forest plot assessing the effects of hyperbaric oxygen in MMSE **(A)**, ADAS-Cog **(B)**, ADL **(C)**, and adverse events **(D)**.

Ten RCTs involving 701 participants were included in the pooled meta-analysis assessing the clinical significance of hyperbaric oxygen compared to the control group on MMSE in AD patients. After excluding one study (Wang et al., 2021) via sensitivity analysis, the heterogeneity diminished from 72 to 20%, and the pooled result illustrated a remarkable improvement in MMSE [MD = 3.08, 95%CI (2.56, 3.61), *I*^2^ = 20%, *p* < 0.00001; Figure 3A]. Three studies (*n* = 218) evaluated ADAS-Cog as an outcome of hyperbaric oxygen intervention, with considerably significant results for the pooled analysis [MD = −4.53, 95%CI (−5.05, −4.00), *I*^2^ = 50%, *p* < 0.00001; Figure 3B].

Additionally, three RTCs (*n* = 244) investigated the effects of hyperbaric oxygen on activities of daily living using ADL, and the pooled analysis revealed significant benefits [MD = 10.12, 95%CI (4.46, 15.79), *p* = 0.0005; Figure 3C], but with heterogeneity across studies (*I*^2^ = 93%). Especially, it was noteworthy that there was no evidence of substantial differences regarding in the outcomes of adverse events between the groups [OR = 1.17, 95%CI (0.68, 2.03), *I*^2^ = 0%, *p* = 0.58; Figure 3D]. The pooled results above are summarized in Table 2.

**Table 2:** The summary results of forest plot for clinical outcomes.

Clinical outcome	Case (n)	OR/SMD/MD 95% CI	*p*	*I^2^* (%)	Model
MMSE	603	3.08 [2.56, 3.61]	<0.00001	20	Fixed
ADAS-Cog	218	−4.53 [−5.05, −4.00]	<0.00001	50	Fixed
ADL	244	10.12 [4.46, 15.79]	0.0005	93	Random
adverse events	468	1.17 [0.68, 2.03]	0.58	0	Fixed
MDA levels	244	−2.83 [−5.27, −0.38]	0.02	98	Random
SOD levels	244	2.12 [1.10, 3.15]	<0.0001	90	Random
IL-1β levels	236	−1.00 [−1.48, −0.53]	<0.0001	66	Random
TGF-β1 levels	158	4.87 [3.98, 5.76]	<0.00001	0	Fixed

#### 3.4.2 Secondary outcomes

**Figure 4:**
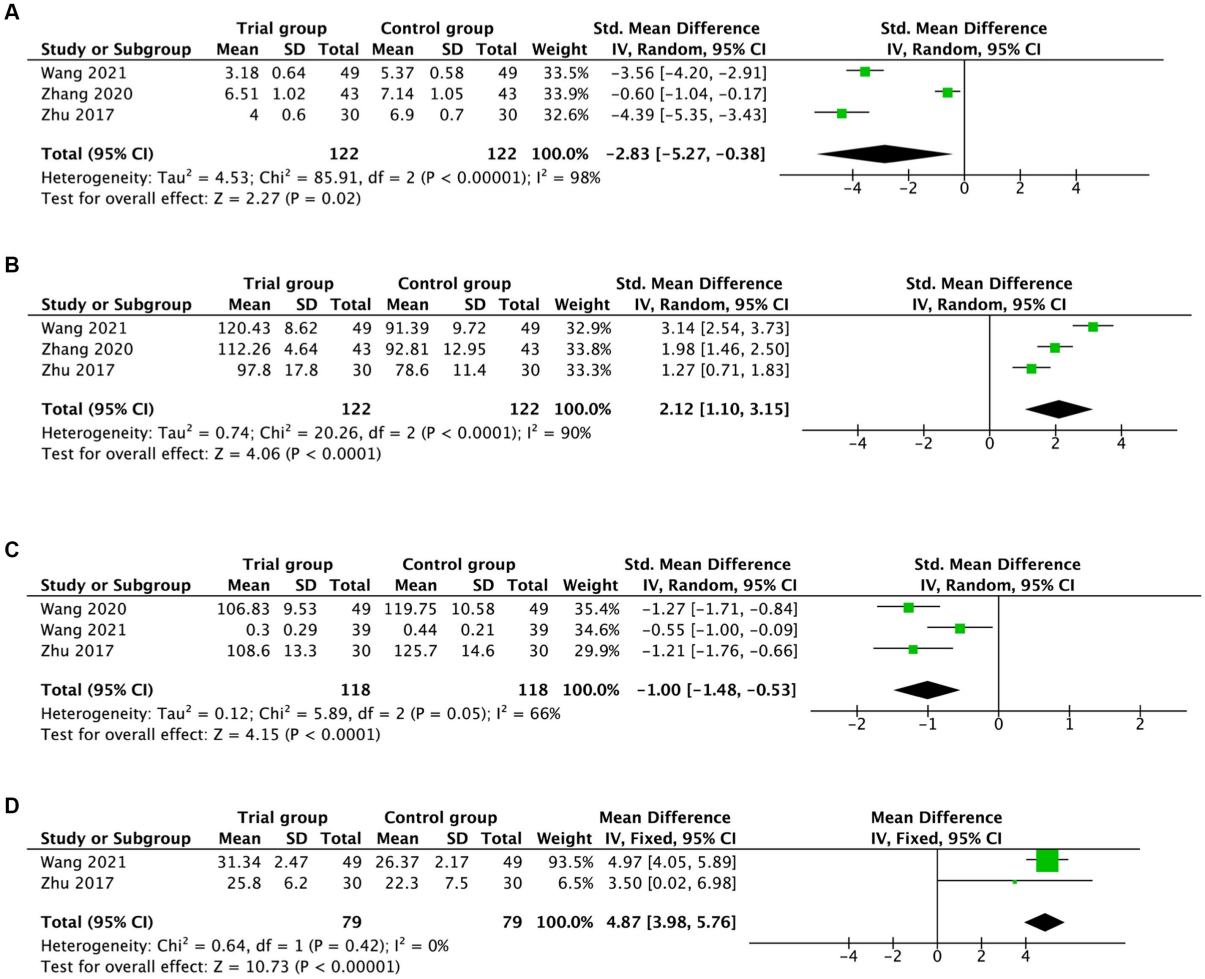
Forest plot assessing the effects of hyperbaric oxygen in serum MDA **(A)**, SOD **(B)**, IL-1β **(C)**, and TGF-β1 **(D)** levels.

Pooling the data gained from blood samples, a statistically remarkable difference in MDA levels was found between the hyperbaric oxygen intervention and the control groups [SMD = −2.83, 95%CI (−5.27, −0.38), *I*^2^ = 98%, *p* = 0.02; Figure 4A]. With regards to SOD levels, the measurements on SOD levels showed significant benefits [SMD = 2.12, 95%CI (1.10, 3.15), *I*^2^ = 90%, *p* < 0.0001, Figure 4B].

Moreover, concerning the IL-1-β levels and TGF-β1 levels, the pooled evidence suggested that hyperbaric oxygen treatment significantly improved IL-1-β levels [SMD = −1.00, 95%CI (−1.48, −0.53), *I*^2^ = 66%, *p* < 0.0001; Figure 4C] and TGF-β1 levels [MD = 4.87, 95%CI (3.98, 5.76), *I*^2^ = 0%, *p* < 0.00001; Figure 4D] in participants with AD. The pooled results above are summarized in Table 2.

## 4 Discussion

Hyperbaric oxygen has been considered as a beneficial intervention for improving cognitive functions and life quality in people with neurodegenerative disorders, which has been proven to involve diverse therapeutic mechanisms. A recent study suggested that hyperbaric oxygen could delay the onset and development of AD via promoting the degradation and clearance of Aβ in the cortices and hippocampi, as well as the levels of C99 produced by β-secretase, the C-terminal fragment of APP, and the C83 fragment levels generated by α-secretase in the brain (Yang et al., 2023). Besides, the expression of p38 MAPK phosphorylation and the levels of TNF-α in the hippocampus were significantly decreased with the help of hyperbaric oxygen, which was closely related with neuronal damage in AD (Zhao et al., 2017). Furthermore, an interesting study utilizing Aβ25-35-induced AD rats indicated that although SOD activity was reduced and MDA content was increased in the hippocampus of AD model rats, the morris water maze test showed that the escape latency was shorter in AD rats than in normal group and these oxidative stress and memory dysfunction related cytokines were significantly ameliorated after hyperbaric oxygen treatment (Tian et al., 2012). Moreover, some investigations also proved that neuronal toxicity and cognitive impairment could be alleviated by hyperbaric oxygen treatment via blocking mitochondria-mediated apoptosis, such as Bax, Bcl-2, Caspase-9, and Caspase-3 in the hippocampus of AD rats (Tian et al., 2013). Similarly, Zhang et al. also found that hyperbaric oxygen played a vital role in reducing apoptosis through NF-κB pathway in the hippocampus, thus facilitating the cognitive, and memory capacities of AD rats (Zhang et al., 2015). Notably, another clinical study based on fluorodeoxyglucose positron emission tomography demonstrated that hyperbaric oxygen intervention could ameliorate cognitive impairment in participants with AD, which might be associated with the improvement of glucose metabolism in brain tissues including the left medial frontal gyrus, right associative visual cortex, right inferior parietal lobule, and so forth (Chen et al., 2020). Simultaneously, cognitive performances like reduced times of calculation and response, along with short-term and working memory in elderly people could also be enhanced. The underlying mechanisms might be related to the increased cerebral blood flow after treatment with hyperbaric oxygen (Shapira et al., 2021).

### 4.1 Main results

This meta-analysis provides some evidence that hyperbaric oxygen intervention has remarkable benefits in improving MMSE, ADAS-Cog, and ADL scores in people with AD. Clinically, MMSE, ADAS-Cog, and ADL were often employed to detect AD and assess therapeutic efficacy with high sensitivity and specificity (Raghavan et al., 2013; Pinto et al., 2019; Saari et al., 2020). In addition, mounting research has suggested that oxidative stress and inflammation were the major factors accompanying the development and progression of AD (Cui et al., 2019; Ren et al., 2023). Diminished SOD levels and elevated MDA levels tended to aggravate neuronal apoptosis and spatial learning and memory deficits (She et al., 2023). Further, the secretion of IL-1-β and TGF-β1 were associated with mitochondrial metabolic activity, immunomodulation as well as neuroprotection in the brain, and thus they could be considered as novel therapeutic targets for the therapy of AD (Cui et al., 2019; Kapoor and Chinnathambi, 2023). Interestingly, this study illustrated that the levels of MDA, SOD, IL-1-β, and TGF-β1 in people with AD could be significantly improved with the help of hyperbaric oxygen treatment. Meanwhile, there was no statistical difference in adverse events (e.g., diarrhea, headache, earache, and insomnia) between the hyperbaric oxygen group and medical treatment group, which was one of the important concerns throughout clinical practice. Simultaneously, people with AD undergoing hyperbaric oxygen were not reported to experience serious adverse events such as middle ear barotrauma, seizure, and pulmonary hemorrhage across studies. Taken together, we consider hyperbaric oxygen treatment to be a valuable option for people with AD, according to the pooled evidence above.

### 4.2 Limitations of this research

However, this meta-analysis has several limitations. First, during the process of records screening, nine RCTs without the reporting of diagnostic criteria for AD were finally excluded according to our stringent inclusion criteria; meanwhile, to reflect the value of hyperbaric oxygen in this unique population with AD, two RCTs mixing AD with vascular dementia were excluded as well, which left us with only 11 studies involved for further analysis. Second, the MMSE, ADAS-Cog, and ADL tests were pooled from different hospitals, but all included studies failed to adopt blinding not only for participants but also for outcomes assessment, which might inevitably lead to subjective deviations in the evaluation. Therefore, more RCTs with high-quality study designs and better methodological descriptions are needed in the future. Third, the disease duration of AD may lead to differences in hyperbaric oxygen efficacy, with shorter disease duration possibly achieving greater improvement. Nevertheless, none of the included RCTs mentioned outcomes based on disease duration. Hence we were not able to assess the clinical significance (such as clinical symptom correlation) of hyperbaric oxygen among different disease duration groups. Fourth, although we did not apply any region restrictions throughout our comprehensive articles identification, only 11 RCTs conducted in China were included. This might make our results unrepresentative of countries outside China. However, we assume that there will be more researchers investigating the clinical value of hyperbaric oxygen for people with AD based on this meta-analysis. Fifth, due to the relatively small sample sizes, we failed to perform Begg’s and Egger’s tests to further detect the potential publication bias. Whereas our results were robust after verifying by sensitivity analysis according to the instructions of the Cochrane Collaboration Handbook.

### 4.3 Implications for future research

First, there is some clinical evidence that hyperbaric oxygen intervention may provide benefits to people with AD. However, several biological factors such as gender, age, education level, and APOE epsilon4 risk gene which may be associated with therapeutic effect were not estimated since the included studies did not provide the related data. Therefore, it will be interesting and rewarding for further research to ascertain the correlation of biological factors with therapeutic effects. Second, in addition to treatment efficacy, the popularized 2nd-level efficacy is also a critical concern throughout our clinical practice. Meanwhile, we have carefully checked the PubMed database to learn about the current large-cohort and large-center plan exploring the popularized 2nd-level efficacy, and the retrieved documents indicate that a lot of studies will be performed in the near future. Hence, there is also a necessity for subsequent meta-analyses in this area to realize the popularized 2nd-level efficacy analysis. Third, recently, a great number of meta-analyses have also revealed that aerobic exercise such as physical activity and exercise interventions could significantly improve cognitive performance and reduce the incidence of AD (Groot et al., 2016; Zhou, 2018; López-Ortiz et al., 2021; Zhang et al., 2023). Therefore, it will be a novel direction to compare the differences between hyperbaric oxygen intervention and aerobic exercise in future research.

## 5 Conclusion

In summary, our results suggest that hyperbaric oxygen intervention can remarkably improve some markers of cognitive function as assessed by MMSE, ADAS-Cog, and ADL tests in the AD population. Meanwhile, the oxidative stress markers (MDA and SOD) and inflammation cytokines (IL-1β and TGF-β1) in blood can also be improved. Still, larger-scale multicenter RCTs with more rigorous designs are needed to further verify the efficacy of hyperbaric oxygen in people with AD.

## Data availability statement

The original contributions presented in the study are included in the article/Supplementary material, further inquiries can be directed to the corresponding author.

## Author contributions

GL: Conceptualization, Data curation, Formal analysis, Methodology, Project administration, Resources, Software, Validation, Visualization, Writing – original draft, Writing – review & editing. LZ: Conceptualization, Data curation, Formal analysis, Funding acquisition, Investigation, Methodology, Writing – original draft. JL: Conceptualization, Project administration, Resources, Software, Supervision, Writing – original draft. XL: Funding acquisition, Methodology, Resources, Validation, Writing – review & editing. LX: Conceptualization, Funding acquisition, Methodology, Project administration, Supervision, Writing – review & editing.
