# Supplementary material for: Clinical evidence of hyperbaric oxygen therapy for Alzheimer’s disease: a systematic review and meta-analysis of randomized controlled trials
Source: Front Aging Neurosci. 2024 Mar 21;16:1360148. doi: 10.3389/fnagi.2024.1360148 (PMC10991696; doi:10.3389/fnagi.2024.1360148)
Supplement: Supplementary file 1 [file Table_1.doc]

**Table 1** The details of hyperbaric oxygen therapy program

| **Study** | **Year** | **Total case (n)** | **Pressure** | **Treatment duration** | **Treatment session** | **Air breaks** | **Timing of outcomes assessment** |
| --- | --- | --- | --- | --- | --- | --- | --- |
| Huang(15) | 2007 | 92 | not applicable | 120 minutes | once a day * not applicable | not applicable | post-treatment |
| Tian(16) | 2007 | 92 | not applicable | 120 minutes | once a day * 3 months | not applicable | post-treatment |
| Yuan(17) | 2010 | 43 | 0.15±0.2 MPa | 120 minutes | once a day * 12 weeks | not applicable | 12 weeks after treatment |
| Liu(18) | 2011 | 80 | 0.2 MPa | 110 minutes | once a day * 45 to 75 days | 10 minutes | six months after treatment |
| Gao(19) | 2017 | 60 | 0.2 MPa | 120 minutes | five time a week * 8 weeks | 2 minutes | eight weeks after treatment |
| Zhu(20) | 2017 | 60 | 0. 23 kPa | 80 minutes | once a day * 2 weeks | 10 minutes | at the end of the last treatment |
| Xu(21) | 2019 | 72 | 0. 2 kPa | not applicable | five time a week * 2 months | 10 minutes | two months after treatment |
| Wang(22) | 2020 | 78 | 0.2 MPa | 80 minutes | five time a week * 6 months | 5 minutes | six months after treatment |
| Zhang(23) | 2020 | 86 | 0.25 MPa | 80 minutes | not applicable * 40 days | 10 minutes | post-treatment |
| Wang(24) | 2021 | 98 | 0. 23 kPa | 80 minutes | once a day * 2 weeks | 10 minutes | two weeks after treatment |
| Zhao(25) | 2021 | 86 | 0.2 MPa | 90 minutes | once a day * 3 months | 10 minutes | post-treatment |
